# Psoriasis outcomes following metabolic bariatric surgery - a systematic review of the literature

**DOI:** 10.1186/s12893-026-03502-1

**Published:** 2026-01-12

**Authors:** Dimitrios Kehagias, Charalampos Lampropoulos, Michael Pellen, Sameh Abogabal, Muhammad Ijlal Haider, Ioannis Kehagias, Prashant Jain

**Affiliations:** 1 Department of Upper Gastrointestinal Surgery, Castle Hill Hospital, Hull University Teaching Hospitals NHS Trust, Hull, UK; 2 Intensive Care Unit, Saint Andrew’s General Hospital of Patras, Patras, Greece; 3 Department of Surgery, University General Hospital of Patras, Rion, Patras, 26504 Greece

**Keywords:** Psoriasis, Metabolic bariatric surgery, Obesity, Metabolic syndrome

## Abstract

**Background:**

Metabolic bariatric surgery (MBS) has been associated with improved clinical outcomes of psoriasis, yet evidence-based guidelines remain unclear. This systematic review aims to analyze current evidence on psoriasis outcomes following MBS and identify factors influencing clinical response.

**Methods:**

A systematic literature search of PubMed®, Scopus, and Google Scholar® was conducted according to PRISMA guidelines. Data extracted included study design, patient demographics, type of MBS, weight loss, and psoriasis outcomes assessed by Psoriasis Area Severity Index (PASI), percentage of affected body surface area (%ABSA), nail involvement, Dermatology Life Quality Index (DLQI), and treatment requirements. Subgroup analysis compared gastric bypass with non-bypass procedures. Due to heterogeneity, a qualitative synthesis was performed.

**Results:**

Six studies involving 447 patients were included. Demographic data were available for 159 patients (mean age 46.9 years, mean BMI 43.8 kg/m²). Pooled BMI reduction was 11.0 ± 3.3 kg/m². Baseline therapies included systemic in 53%, topical in 44%, and phototherapy in 5%. After surgery, systemic therapy decreased to 34%, topical to 39%, and 29% required no treatment. DLQI decreased from 14.9 to 5.0, %ABSA from 5.7 to 1.7, PASI from 3.6 to 1.2, and nail involvement from 43.8% to 21.9% (p < 0.05). Overall, 69.5% of individuals demonstrated improvement or remission of psoriasis, primarily defined by reduced or discontinued treatment. Improvement or remission was noted in 80% of gastric bypass and 10% after non-bypass surgery, with higher PASI reduction after bypass (ΔPASI 2.32 vs 1.67). Factors related to improved clinical response included greater excess weight loss, older age, Roux-en-Y gastric bypass and absence of family history of psoriasis.

**Conclusions:**

MBS may improve psoriasis severity, quality of life, and treatment requirements, but current evidence is uncertain. Weight loss magnitude, type of MBS and patient characteristics might affect clinical response of psoriasis. Further prospective studies are required to establish personalized management among individuals with psoriasis and obesity.

**Trial registration:**

Registration of systematic review: PROSPERO database (UIN: CRD420251108229)

## Background

Psoriasis is a multisystem inflammatory disorder with autoimmune pathogenesis, affecting approximately 3% of the adult population in the United States [1]. It is frequently associated with cardiovascular disease, metabolic syndrome, obesity, hypertension, type 2 diabetes mellitus (T2DM), and dyslipidemia. In a process termed the ‘psoriatic march’, skin inflammation in psoriasis can further progress to systemic inflammation, and consequently contribute to insulin resistance and endothelial dysfunction [2]. Obesity can further amplify this inflammatory cascade, exacerbating the risk of these comorbidities [3, 4].

Despite accumulating evidence, the role of weight loss in psoriasis management remains underemphasized, limiting patient awareness. According to the latest psoriasis management guidelines published in 2023 by the Dermatological Society of Singapore, counseling obese and overweight patients on the importance of weight loss for improving treatment response, carries a grade D recommendation, supported by level 2–3 evidence [5]. Therefore, it is mandatory to acquire more evidence, raise awareness about the link of psoriasis and excess weight, and inform tailor-made approaches for optimal outcomes.

Improvement in psoriasis may occur before substantial weight loss, suggesting mechanisms beyond caloric reduction, including changes in glucagon-like peptide-1 (GLP-1) secretion [6]. Several studies have reported an association between GLP-1 receptor agonists (GLP-1 RAs) and psoriasis improvement in patients with obesity and T2DM, and three meta-analyses have demonstrated that GLP-1 RAs significantly reduce psoriasis severity [7–10]. Despite these findings, the 2021 Joint American Academy of Dermatology–National Psoriasis Foundation (NPF) guidelines state that the role of GLP-1 RAs in psoriasis management remains unclarified and, consequently, they are not currently recommended [11]. Metabolic bariatric surgery (MBS), in contrast, induces more robust and sustained weight loss and hormonal changes, including rapid increases in GLP-1, which may contribute to reductions in systemic inflammation and improvements in psoriasis severity. Comparative studies indicate that MBS results in superior long-term weight loss and glycemic control compared with non-surgical interventions such as semaglutide and tirzepatide, and is associated with lower all-cause mortality in patients with obesity and T2DM [12, 13].

In relation to psoriasis, several studies have reported clinical improvement following MBS, although the underlying mechanisms remain unclear. The first documented case, published in 2004, described a patient with obesity and a 15-year history of psoriasis who achieved complete remission for two years after undergoing laparoscopic Roux-en-Y gastric bypass (RYGBP) [14]. In the following years, further evidence was furnished from reports and systematic reviews, indicating that MBS may have beneficial outcomes in patients with psoriasis [15, 16].

Despite these observations, most studies have focused on evaluating GLP-1 RAs for the management of psoriasis in patients with obesity, whereas evidence regarding the role of MBS remains limited. The absence of practical guidelines from the American Society for Metabolic and Bariatric Surgery (ASMBS) and the International Federation for the Surgery of Obesity and Metabolic Disorders (IFSO) on the management of patients with concomitant obesity and psoriasis, further contributes to this gap. The aim of this systematic review is to synthesize the available data on psoriasis outcomes following MBS and to identify peri-operative factors associated with improved clinical response.

## Methods

### Study design

A systematic review of the literature was conducted, in accordance with the Preferred Reporting Items for Systematic Reviews and Meta-Analyses (PRISMA) guidelines and was registered in the PROSPERO database (UIN: CRD420251108229) [17]. Google Scholar^®^, Scopus and PubMed^®^ (National Library of Medicine, Bethesda, MD, USA) were searched for articles published until July 2025. With Google Scholar^®^ we aimed to retrieve grey literature and studies not indexed in traditional databases.

The search strategy included a combination of Medical Subject Headings (MeSH) terms, with Boolean operators. The search syntax was: ((psoriasis) AND (psoriasis area severity index OR PASI OR nail psoriasis severity index OR NPSI)) AND (sleeve gastrectomy OR SG OR Roux-en-Y gastric bypass OR RYGBP OR bariatric surgery). The registered protocol in PROSPERO includes the advanced search syntax. Three authors performed the literature search. Duplicate articles were removed prior to screening. Following that, titles and abstracts were reviewed and irrelevant studies were excluded.

The retrieved articles were rigorously evaluated for eligibility. Studies were eligible if they reported outcomes of individuals with psoriasis and obesity after MBS. Inclusion criteria were as follows: (1) articles in English language, (2) cohort studies, and (3) case series. The exclusion criteria were: (1) publications not in English language, (2) short commentaries, or conference abstracts, (3) case reports, (4) books or chapters, (5) reviews, and (6) studies reporting outcomes of psoriasis after non-surgical weight-loss interventions.

### Data extracted and definitions

From each eligible study, the following information was extracted: study characteristics (first author, year of publication, country, and study design), patient demographics (age, sex, body mass index [BMI]), and comorbidities when reported. Surgical variables included the type of MBS performed and the reported follow-up for psoriasis outcomes assessment.

Weight-loss outcomes were collected as reported by the individual studies. These included excess weight loss (EWL%), change in BMI (ΔBMI), and total body weight loss (TBWL%). Standardized definitions were applied:

- EWL% = (postoperative weight loss)/(preoperative excess weight at time of surgery) × 100.
- TBWL% = [(initial weight) – (postop weight)]/[(initial weight)] × 100.
- ΔBMI = preoperative BMI - postoperative BMI.

Family history of psoriasis, presence of psoriatic arthritis, and disease duration prior to surgery were extracted from the studies. Psoriasis outcomes were assessed based on changes in medication use and severity indices. Treatment modalities were categorized as systemic therapy (cyclosporine, methotrexate, biologics), topical therapy (corticosteroids, calcineurin inhibitors, vitamin D analogues, tazarotene, salicylic acid, emollients), or phototherapy.

Psoriasis was assessed with the following clinical measures:

- Percentage of affected body surface area (%ABSA): estimated using the National Psoriasis Foundation’s palm method, in which the surface of the patient’s palm corresponds to 1% of body surface [18].
- Psoriasis Area and Severity Index (PASI): a composite score evaluating erythema, induration, and scaling in four regions (head, trunk, upper extremities, lower extremities), graded on a 0–4 scale. Scores were calculated by multiplying severity ratings by the proportion of body surface involved in each region, with the sum representing the final PASI score. PASI categories were defined as mild (<5), moderate (5–10), and severe (>10) [19].
- Dermatology Life Quality Index (DLQI): a 10-item questionnaire assessing the impact of skin disease on health-related quality of life. Scores range from 0 to 30, with values >10 indicating a very large or extremely large effect on quality of life [20].
- Nail involvement: The nail psoriasis severity index is used to assess the severity of nail bed psoriasis and nail matrix psoriasis by area of involvement in the nail unit [21].

### Data synthesis

Substantial clinical and methodological heterogeneity was observed among the included studies, primarily due to variations in outcome definitions and measures of psoriasis response following MBS. This precluded the performance of a meta-analysis. Consequently, a qualitative synthesis of the evidence was undertaken.

Quantitative variables were documented as mean ± standard deviation (SD). When studies presented data as median ± interquartile range (IQR), values were converted to mean ± SD. When feasible, mean ± SD values were summarized descriptively across studies to provide an overview of central tendency. These pooled values represent simple descriptive calculations and should not be interpreted as inferential estimates. Psoriasis outcomes were classified into three categories: remission, no change, and worsening. Because definitions for ‘complete’ and ‘partial’ remission varied substantially across studies and were not supported by standardized criteria, these categories could not be reliably distinguished. For consistency and comparability, remission was therefore synthesized as a single category (encompassing both partial and complete remission), alongside ‘no change’ and ‘worsening.’ Remission encompassed both complete remission or discontinuation of treatment after MBS, and partial remission or decrease in treatment requirements. In studies that did not clarify whether psoriasis was stable or worsened, this was presented as one category.

To enhance interpretability, a subgroup analysis was performed according to surgical type. Procedures were categorized as gastric bypass [RYGBP, one-anastomosis gastric bypass (OAGB)] or non-bypass [sleeve gastrectomy (SG), adjustable gastric banding (AGB)]. Outcomes were extracted when reported and summarized in tabular format.

Potential factors associated with improved psoriasis outcomes were also recorded. When studies applied statistical analyses, significant associations (*p* < 0.05) were extracted and presented separately. Supporting evidence from each study was also documented in a separate column.

### Quality assessment

Two authors assessed the quality and risk of bias of the included studies. For case series the Joanna Briggs Institute (JBI) critical appraisal tool was applied [22]. For the observational studies the ROBINS - I V2 tool was utilized [23]. A third author was involved for resolving any disagreements during the process.

## Results

### Study characteristics

**Fig. 1:**
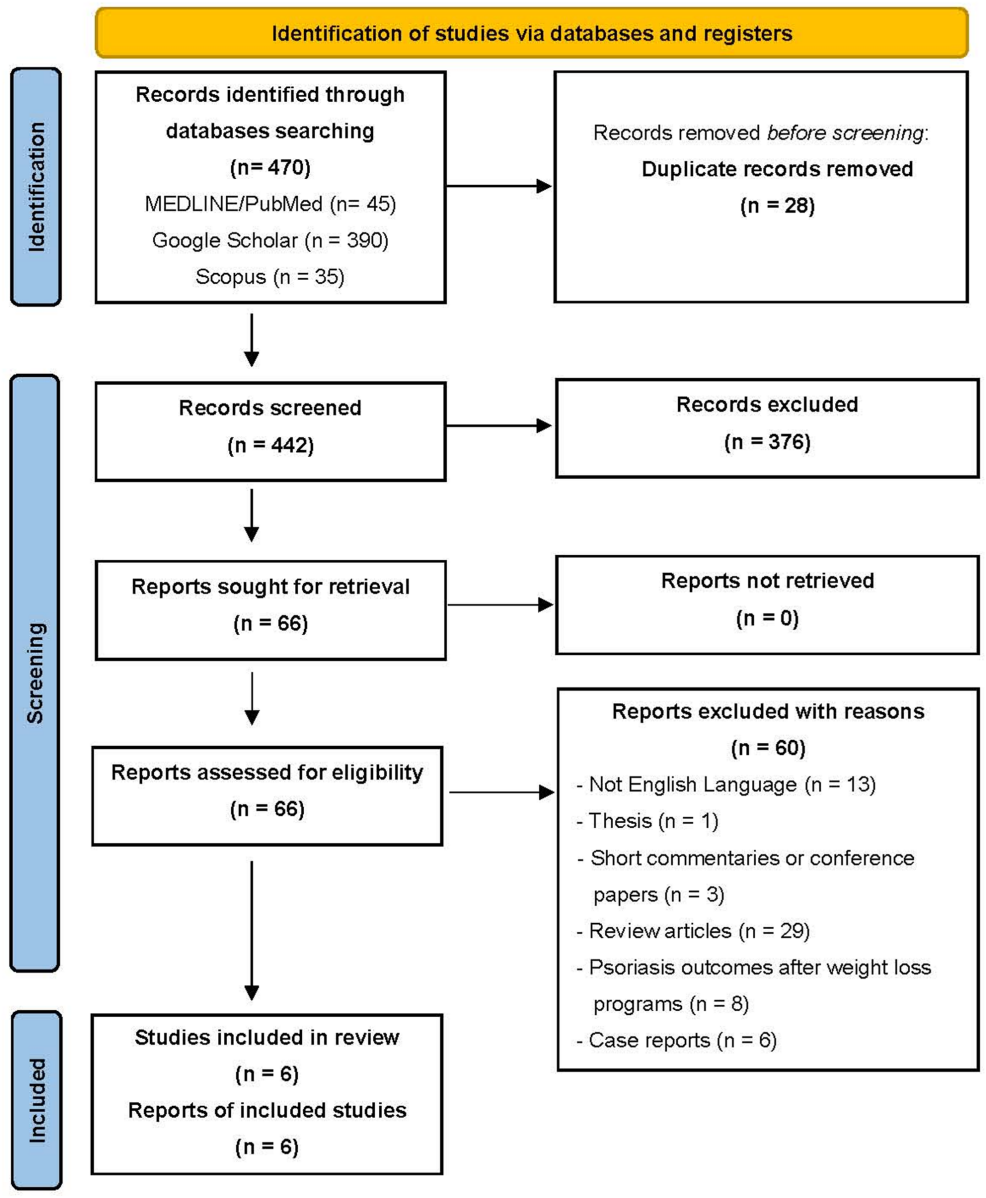
PRISMA Flowchart of studies assessing psoriasis outcomes after metabolic bariatric surgery

After the screening process and applying the inclusion and exclusion criteria, six studies published between 2012 and 2024, met the inclusion criteria and were analyzed (Fig. 1) [24–29]. Two studies originated from the United States, two from Scandinavian countries, one from Chile, and one from Iran. Study designs included three case series, two population-based cohort studies, and one prospective cohort study.

In total, 447 patients with psoriasis who underwent MBS were included. Demographic data were available for 159 patients across five studies. Among them, 42 (26.4%) were male and 117 (73.6%) females, with a mean age of 46.9 ± 10.2 years and mean BMI of 43.8 ± 8.5 kg/m². Comorbidities were reported in three studies, including T2DM, hypertension, and dyslipidemia.

Data regarding the procedure were available for all patients: 382 underwent RYGBP, 13 underwent OAGB, 27 SG, and 24 AGB, while in one patient the procedure was unknown. Reported follow-up for psoriasis outcomes ranged from 6 months to 5 years. Family history of psoriasis was reported in three studies (35/99 patients, 35.3%), psoriatic arthritis in four studies (96/403 patients, 23.8%), and psoriasis duration in two studies (20–24 years) (Table 1).

**Table 1:** Study characteristics including patients with psoriasis that underwent metabolic bariatric surgery

Author	Year	Country	Study design	Patients with psoriasis (*n*)	Age (y)	Sex	BMI (kg/m^2^)	Comorbidities	Family history of psoriasis	Psoriatic arthropathy	Time with psoriasis (y)	Type of bariatric surgery	Follow-up for psoriasis (months)
Farias et al.	2012	Chile	Case series	10	41.2 ± 13	M (2) F (8)	38.8 ± 5.2	HTN (7) T2DM (4)	.		.	RYGBP (8) SG (2)	6–30
Hossler et al.	2013	USA	Case series	34	49.8 ± 10.7	M (4) F (30)	48.5 ± 8.5	.	17/34 (50%)		20 ± 16.8	RYGBP (30) AGB (3) Unknown (1)	.
Romero – Talamas et al.	2014	USA	Case series	33	50.8 ± 10.0	M (13) F (20)	50.2 ± 10.1	HTN (23) T2DM (21) DLD (25)	3/33 (9.1%)	8/33 (24.2%)	24 ± 9	RYGBP (21) SG (8) AGB (4)	26.2 ± 20.3
Egeberg et al.	2017	Denmark	Retrospective population-based cohort study	288	.	.	.	.	.	59/288 (20.4%)	.	RYGBP (272) AGB (16)	.
Laskowski et al.	2021	Sweden	Retrospective nationwide registry study	50	43.8 ± 7.7	M (20) F (30)	39 ± 5.04	.	.	15/50 (30%)	.	RYGBP (40) SG (9) AGB (1)	24
Hosseininasab et al.	2024	Iran	Prospective cohort study	32	46.6 ± 10.8	M (3) F (29)	41.5 ± 4.7	HTN (4) T2DM (4) DLD (7)	15/32 (46.9%)	14/32 (43.8%)	.	RYGBP (11) OAGB (13) SG (8)	70.6 ± 29.1

*HTN* hypertension, *T2DM* type 2 diabetes mellitus, *DLD* dyslipidemia, *RYGBP* Roux-en-Y gastric bypass, *SG* sleeve gastrectomy, *AGB* adjustable gastric banding, *OAGB* one-anastomosis gastric bypass

* Values are expressed as mean ± SD

**Fig. 2:**
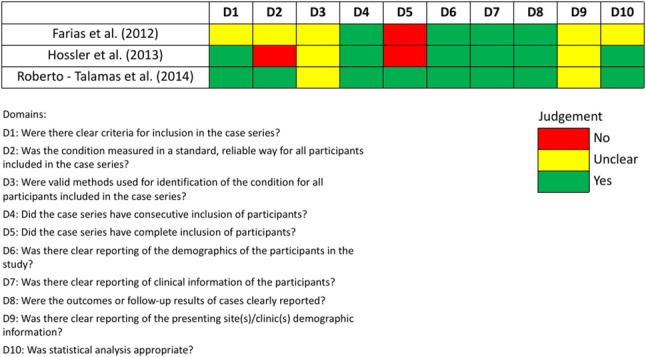
Risk of bias assessment for case series using JBI critical appraisal tool

**Fig. 3:**
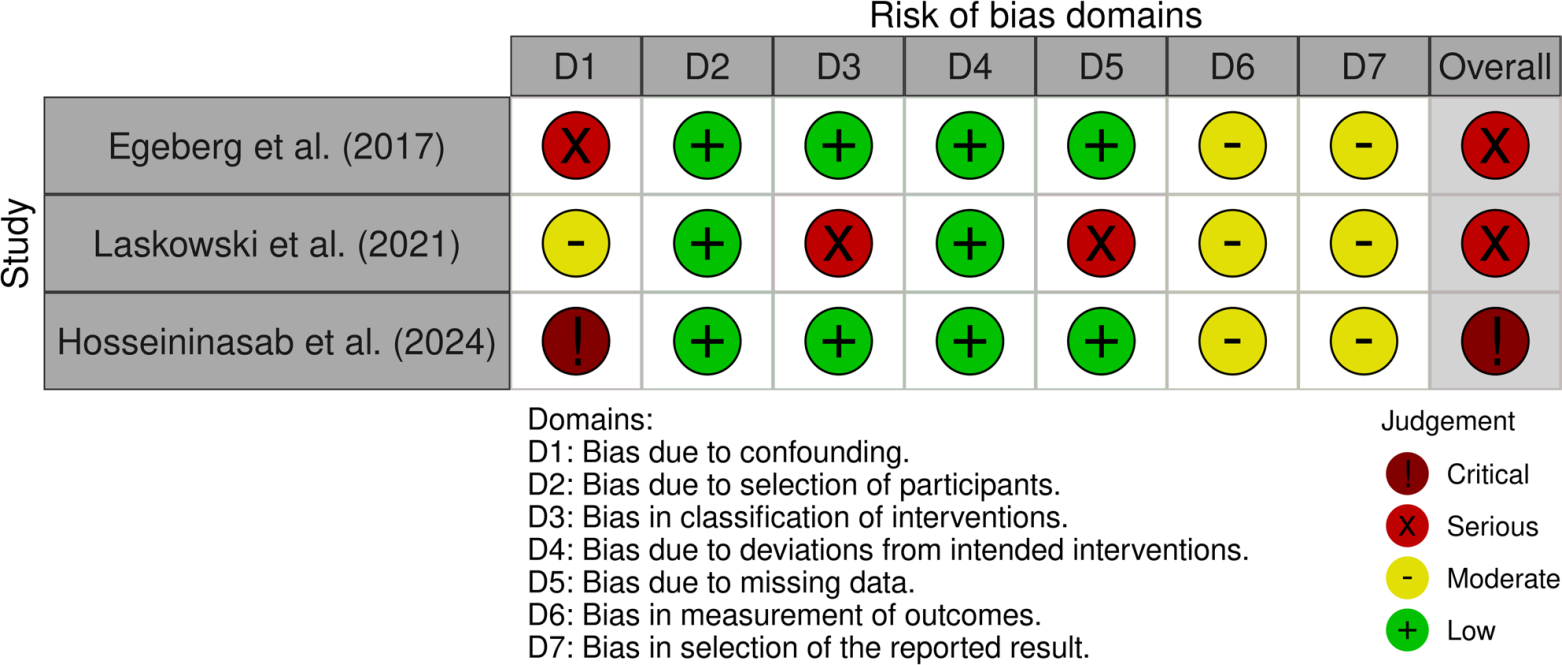
Risk of bias assessment for observational studies using the ROBINS-I V2 tool

Among the three case-series assessed with the JBI critical appraisal tool, one was rated as good quality, while the remaining two were of moderate quality (Fig. 2). In contrast, of the observational studies, two were judged to have a serious risk of bias and one was classified as having a critical risk of bias (Fig. 3).

### Weight loss and comorbidities resolution

Weight loss outcomes and comorbidity resolution were reported in five and two studies, respectively (Table 2). EWL% was assessed in three studies, ranging from 48.7 to 88%. TBWL% was documented in one study (27.6 ± 11.5%), while ΔΒΜΙ was available in four studies (149 patients), with a pooled mean reduction of 11.01 ± 3.3 kg/m^2^ [25, 26, 28, 29]. In the two studies reporting comorbidity resolution, T2DM resolved in 75% and 61.5% of patients, hypertension in 57% and 51.6%, and dyslipidemia (reported in one study) in 78.6% [24, 26].

**Table 2:** Weight loss and comorbidities resolution after metabolic bariatric surgery

Author	Weight loss			Comorbidities resolution		
	EWL %	ΔBMI	TBWL %	T2DM (%)	Hypertension (%)	Dyslipidemia (%)
Farias et al.	88.2	.	.	3/4 (75)	4/7 (57)	.
Hossler et al.	.	12.3 ± 2.04	.	.	.	.
Romero – Talamas et al.	48.7 ± 26.6	12.3 ± 2.43	.	13/21 (61.5)	12/23 (51.6)	20/25 (78.6)
Laskowski et al.	.	8.97 ± 1.07	.	.	.	.
Hosseininasab et al.	72.8 ± 30.6	11.5 ± 5.4	27.6 ± 11.5	.	.	.

*EWL %* excess weight loss, *ΔBMI* difference of baseline and follow-up body mass index, *TBWL* total body weight loss, *T2DM* type 2 diabetes mellitus

Values expressed as mean ± SD

### Psoriasis outcomes

Five studies reported changes in psoriasis treatment after MBS [24–26, 28, 29]. Four of them assessed clinical measures, including DLQI, %ABSA, PASI, and nail involvement [24, 26, 28, 29]. One nationwide registry study reported only remission of psoriasis after MBS, without assessing clinical indices [27].

Among 159 patients across five studies, baseline treatment included systemic medications in 85 patients (53.4%), topical therapy in 70 (44%), phototherapy in 5, and no treatment in 9 (5%). In the study of Hosseininasab et al. some received combination therapy. Postoperatively, 54 patients remained on systemic treatment (33.9%), 62 on topical therapy (38.9%), none on phototherapy, and 46 (28.9%) required no treatment.

Three of four studies assessing clinical outcomes demonstrated significant improvement. DLQI decreased significantly in one study, from 14.9 ± 6.8 to 5 ± 6.3 (*p* < 0.05), while another study showed not significant reduction, from 2.67 ± 5.34 to 2 ± 1.53 [24, 28]. %ABSA decreased significantly from 5.7 ± 4.1 to 1.7 ± 2.1 (*p* < 0.05) [26]. PASI score decreased in two studies, with one reporting a significant reduction, from 3.6 ± 5.90 to 1.20 ± 3.45 (*p* < 0.05) [28, 29]. Nail involvement decreased significantly in one study from 43.8% to 21.9% [29].

Overall, of 447 patients (69.5%) demonstrated psoriasis improvement or remission, defined as reduction or discontinuation of psoriasis-directed therapy, as reported in the included studies. In studies distinguishing stable and worsening disease, only 9 of 109 patients (8%) experienced worsening (Table 3).

**Table 3:** Psoriasis characteristics at baseline and outcomes at follow-up

Author	Follow-up duration (months)	Parameters	Baseline	Follow-up	Outcomes		
					Improvement or remission	No changes	Worse
Farias et al.	6–30	n	10	10	7/10	2/10	1/10
		Treatment	Systemic drugs (4) Topical (5) No treatment (1)	Systemic drugs (1) Topical (5) No treatment (4)			
		DLQI	14.9 ± 6.8	5 ± 6.3 *			
Hossler et al.	.	n	34	34	21/34	9/34	4/34
		Treatment	Systemic drugs (10) Topical (22) None (2)	Systemic (6) Topical (19) None (9)			
Romero – Talamas et al.	26.2 ± 20.3	n	33	33	13/33	19/33	1/33
		Treatment	Systemic (9) Topical (24)	Systemic (5) Topical (20) None (8)			
		%ABSA	5.7 ± 4.1	1.7 ± 2.1 *			
Egeberg et al.	.	n	288	.	224/288	64/288	
Laskowski et al.	24	n	50	Treatment (50/50) PASI (28/50) DLQI (26/50)	25/50	25/50	
		Treatment	Systemic (44) None (6)	Systemic (25) None (25)			
		PASI	5.8 ± 6.03	3.37 ± 4.81			
		DLQI	2.67 ± 5.34	2 ± 1.53			
Hosseininasab et al.	70.6 ± 29.1	n	32	32	21/32	8/32	3/32
		Treatment	Systemic (18) Topical (19) Phototherapy (5)	Systemic (17) Topical (18) Phototherapy (0)			
		PASI	3.6 ± 5.90	1.20 ± 3.45 *			
		Nail involvement	14/32 (43.8%)	7/32 (21.9%) *			

*DLQI* Dermatology Life Quality Index, *%ABSA* affected body surface area, *PASI* psoriasis area and severity index

Values expressed as mean ± SD, * *p* < 0.05

### Subgroup analysis by procedure type

Four studies stratified psoriasis outcomes by MBS type [24, 26, 27, 29]. A total of 325 patients underwent gastric bypass (RYGBP or OAGB) and 38 underwent non-bypass procedures (SG or AGB). Hosseininasab et al. reported greater PASI reduction after gastric bypass compared to non-bypass surgery (ΔPASI 2.32 ± 4.32 vs. 1.67 ± 3.31) [29].

In the other three studies, 241 of 301 gastric bypass patients (80%) experienced improvement or remission, while 20% remained stable or worsened [24, 26, 27]. Conversely, among 30 patients who underwent non-bypass procedures, only 3 (10%) experienced remission. Two (6.6%) worsened, 10 (33.4%) remained stable, and 50% experienced either stable or worsening disease (Table 4).

**Table 4:** Gastric bypass versus non-gastric bypass in psoriasis outcomes

Author	Number of patients	Gastric bypass (*n* = 325)				Non gastric bypass (*n* = 38)			
		Improvement Remission	Unchanged	Worsening	ΔPASI	Improvement Remission	Unchanged	Worsening	ΔPASI
Farias et al.	SG (2) RYGBP (8)	7/8	1/8	.	.	.	1/2	1/2	.
Romero – Talamas et al.	SG (8) RYGBP (21) AGB (4)	11/21	10/21	.	.	2/12	9/12	1/12	.
Egeberg et al.	RYGBP (272) AGB (16)	223/272	49/272		.	1/16	15/16		.
Hosseininasab et al.	RYGBP (11) OAGB (13) SG (8)	.	.	.	2.32 ± 4.32	.	.	.	1.67 ± 3.31

*SG* Sleeve gastrectomy, *RYGBP* Roux-en-Y gastric bypass, *ΔPASI* difference of PASI score from baseline and postoperative

### Factors associated with improved psoriasis outcomes

Greater %EWL, older age at surgery, absence of family history of psoriasis, and RYGBP were associated with improved clinical response of psoriasis. Specifically, older age was significant in two studies (*p* < 0.05), absence of family history in one, greater %EWL in one, and RYGBP in one (though three other studies found no significant association) [24–27, 29]. One study also suggested that mild baseline psoriasis severity (PASI < 5) was associated with greater benefit [29] (Table 5).

**Table 5:** Factors associated with improved psoriasis outcomes after metabolic bariatric surgery

Author	Factors associated with improved outcomes	Supporting information
Farias et al.	RYGBP	“Related to the surgical technique, RYGBP had better skin outcomes than SG.”
Hossler et al.	Older age at surgery *	Older age at surgery was significantly associated with improved outcomes (52.7 vs. 38.5 years, *p* = 0.039)
	Not family history of psoriasis *	Family history of psoriasis was less likely to report improvement compared with all others (*p* = 0.007)
Romero – Talamas et al.	Older age at surgery *	Older age at the time of surgery (54.8 ± 8.1 versus 48.1 ± 10.4 years, *p* = 0.047)
	RYGBP *	RYGBP vs. non-bypass procedures (52.4% vs. 16.7%, *p* = 0.043)
	Greater EWL% *	Greater %EWL (64.2 ± 26.0 vs. 43.4 ± 23.6, *p* = 0.036)
Egeberg et al.	RYGBP	HRs of psoriasis were 0.52 (95%CI, 0.33–0.81) and 1.23 (95%CI, 0.40–3.75) for RYGBP and AGB. HRs of progression to severe psoriasis were 0.44 (95%CI, 0.23–0.86) and 1.18 (95%CI, 0.12–11.49) for RYGBP and AGB
Hosseininasab et al.	Mild severity of psoriasis (PASI < 5)	Patients with mild psoriasis predominantly experienced beneficial outcomes (12/18, 66.7%)
	RYGBP or OAGB	OAGB and RYGBP showed more significant decrease of PASI score

*RYGBP* Roux-en-Y gastric bypass, *SG* sleeve gastrectomy, *EWL* excess weight loss, *HR* hazard ratio, *AGB* adjustable gastric banding, *PASI* psoriasis area severity index, *OAGB* one anastomosis gastric bypass

* *p* < 0.05, statistically significant

## Discussion

Obesity is a well-established high-risk factor for psoriasis [15, 30]. Weight reduction, in turn, has been shown to improve psoriasis outcomes and may even exert a protective effect against disease development [31]. Among weight-loss strategies, MBS achieves the most durable results in terms of both weight reduction and glycemic control compared with lifestyle interventions or pharmacotherapy, making it a promising option for psoriasis management [32]. Findings from this systematic review may support this concept. Overall, 69.5% of patients experienced improvement or remission of psoriasis, while systemic treatment was decreased from 53% to 34%. Improvements were also noted in DLQI, %ABSA, PASI, and nail involvement, with three of six studies reporting statistically significant results. These findings might suggest that MBS confers meaningful benefits across both clinical outcomes and treatment requirements. However, a key limitation of this review is the overall methodological quality of the included studies. Most of the observational designs carried serious or critical risks of bias. Consequently, the external validity and applicability of these findings are restricted.

Weight-loss interventions have been associated with reduction in psoriasis severity. A meta-analysis of seven randomized clinical trials, concluded that non-surgical, non-pharmacologic weight-loss interventions decrease PASI, with a pooled mean difference (MD) of − 2.49 over a 6-month period [33]. Similarly, another meta-analysis of four prospective and two randomized studies, involving 63 patients treated with GLP-1 receptor agonists, reported a greater reduction in PASI scores, with a pooled MD of − 5.83 after 2–3 months of follow-up [10]. In contrast, studies included in this review reported PASI reductions of − 2.43 at 24 months and − 2.4 at 70 months, among patients that underwent MBS [28, 29]. While GLP-1 RAs have been associated with reductions in PASI scores over short-term follow-up, MBS may provide more sustained improvements in weight, metabolic parameters, and psoriasis outcomes. However, differences in study populations, follow-up durations, and outcome definitions limit direct comparisons, and these observations should be interpreted as hypothesis-generating rather than definitive evidence of superiority.

Apart from the beneficial clinical outcomes, MBS appears to decrease the treatment needs for psoriasis. Obesity has been consistently associated with poorer therapeutic response. A meta-analysis of 40 studies involving 21,438 patients demonstrated that obesity (BMI > 30 kg/m²) was negatively associated with treatment response and PASI reduction (OR 0.57; 95% CI, 0.48–0.66) [34]. In line with this, the 2020 British Association of Dermatologists guidelines on biologic therapy for psoriasis strongly recommend addressing modifiable factors, such as obesity, when biologic agents fail [35]. In our systematic review, the need for systemic medication decreased from 53% to 34%, topical therapy from 44% to 39%, and phototherapy from 5% to 0%. Systemic agents included methotrexate, oral corticosteroids, and biologics (e.g., TNF-α inhibitors). Two studies assessed the use of biologic agents following MBS and presented divergent results. Laskowski et al. reported a reduction in biologic use from 36% to 18% at short-term follow-up, a change not observed in the control group. Although no significant improvements in PASI or DLQI were detected, the early discontinuation of biologics suggests a potential amelioration of psoriasis severity [28]. Conversely, Hosseininasab et al. observed a two-fold increase in biologic use (from 12.5% to 25%), despite an overall reduction in treatment requirements [29]. Collectively, these results indicate that MBS may decrease overall treatment requirements and, in some cases, improve responsiveness to biologic agents, potentially allowing disease control with less intensive regimens.

Surgical type may also influence outcomes. In this review, improvement or remission occurred in 80% of patients who underwent gastric bypass (RYGBP or OAGB), compared with only 10% after non-bypass procedures (SG or AGB). Although none of the included studies directly compared procedures, these findings suggest a possible advantage for gastric bypass. Mechanistically, RYGBP and OAGB induce marked postprandial secretion of incretins, particularly GLP-1, which improves glycemic control even before weight loss is achieved [36]. Psoriasis appears to follow a similar pattern, with clinical improvement reported prior to substantial weight loss, suggesting overlapping mechanisms [6, 37, 38]. GLP-1 RAs appear to inhibit the NF-κB signaling pathway, and via immunomodulatory actions, reduce systemic inflammation and improve PASI scores [39]. However, SG is also related with increased postprandial secretion of GLP-1 via rapid gastric emptying [40], but the evidence to date more strongly supports bypass procedures. None of the included studies were designed for direct comparative evaluation of surgical techniques, and the number of patients undergoing sleeve gastrectomy or adjustable gastric banding was extremely limited. As such, any apparent differences between procedures are exploratory, may be influenced by selection and reporting biases, and should be considered hypothesis-generating rather than conclusive. Prospective, head-to-head studies are required to establish whether bypass truly confers superior benefits for psoriasis outcomes.

Psoriasis outcomes after MBS appear to be influenced by several factors. In this systematic review, older age at surgery, absence of a family history of psoriasis, greater %EWL, and undergoing RYGBP were associated with more favorable outcomes. While the benefit of gastric bypass can be explained by the metabolic and hormonal mechanisms previously discussed, the association between older age and improved outcomes is less clear. Intuitively, one might expect advanced age to negatively impact results due to the chronicity of established disease; however, two studies demonstrated the opposite, suggesting a mechanism that remains to be elucidated [25, 26]. Genetic predisposition also seems relevant. Hossler et al. reported that hereditary psoriasis may be less responsive to weight loss interventions [25]. Similarly, the preoperative severity of psoriasis may influence postoperative outcomes [29]. Additional factors that may contribute include smoking status, prior exposure to biologic agents, and the degree of obesity, although current evidence is limited and based on small sample sizes [34].

Several important limitations need to be acknowledged. A meta-analysis was not able to be performed due to poor quality of study design, and significant heterogeneity across the studies, both clinical and methodological. In particular, different clinical outcome measures were used in each study, while no comparator groups were included. The pooled averages presented in this review reflect descriptive summaries only and do not constitute results from a formal random-effects model, requiring cautious interpretation. Follow-up periods across the included studies ranged from 6 months to 5 years. Given that psoriasis is a chronic, relapsing-remitting disease, pooling outcomes across these variable follow-up durations makes it difficult to determine whether observed improvements are transient or sustained over the long term. This represents an additional limitation of the current evidence base. Moreover, most studies were retrospective, single-center analyses, and case series raising concerns about selection bias and suboptimal study design. Although the review includes studies of different designs, we were unable to conduct meaningful stratified analyses due to the very small number of eligible studies and the substantial methodological limitations across all of them. As a result, the synthesis presented here should be interpreted as a descriptive overview of the available evidence rather than a design-adjusted comparison. Prospective studies with bigger sample size and standard clinical endpoints are required to obtain more reliable results. Additionally, none of the studies directly compared the outcomes between different types of MBS. Beyond clinical observations, mechanistic studies are also needed to clarify the role of gastrointestinal hormones, such as GLP-1, in mediating psoriasis improvement after MBS. Although some studies reported partial versus complete remission separately, the lack of standardized definitions and inconsistent application of cut-offs prevented meaningful distinction between these categories. As a result, remission was analyzed as a combined outcome, which may obscure important clinical differences and should be interpreted with caution. This approach reflects the preliminary and heterogeneous nature of the current evidence and reinforces the need for standardized outcome definitions in future research. While several prior systematic reviews have examined similar research questions, the present review was not intended to supersede these studies but rather to complement them by incorporating subgroup analyses according to surgical technique and by providing additional insights into the relationship between weight loss and psoriasis outcomes. Importantly, MBS should not be considered a therapeutic option for psoriasis; however, in patients with psoriasis and concomitant obesity who meet established indications for surgery, improvement in disease severity may occur as a secondary benefit mediated by weight loss and reduction in systemic inflammation. The assessment of surgical risks and adverse events, which are well described in the bariatric literature and must be carefully weighed in clinical decision-making, was beyond the scope of the present review.

## Conclusions

MBS might be related with beneficial outcomes regarding clinical measures, quality of life, and treatment requirements among individuals with obesity and psoriasis, but the current evidence is uncertain. Type of MBS, age at surgery, weight loss outcomes and family history of psoriasis could affect clinical response. Despite the uncertainty of the available data, preliminary findings suggest a positive association between MBS and psoriasis outcomes, supporting the need for well-designed, large, prospective, controlled studies to provide definitive evidence.

## Data Availability

The datasets used and analyzed during the current study are available from the corresponding author on reasonable request.

## Abbreviations

MBS: Metabolic bariatric surgery
PASI: Psoriasis Area Severity Index
%ABSA: percentage of affected body surface area
DLQI: Dermatology Life Quality Index
T2DM: type 2 diabetes mellitus
GLP-1: glucagon-like peptide-1
GLP-1 Ras: GLP-1 receptor agonists
IL: interleukin
TNF: tumor necrosis factor
RYGBP: Roux-en-Y gastric bypass
ASMBS: American Society for Metabolic and Bariatric Surgery
IFSO: International Federation for the Surgery of Obesity and Metabolic Disorders
PRISMA: Preferred Reporting Items for Systematic Reviews and Meta-Analyses
BMI: body mass index
EWL: excess weight loss
TBWL: total body weight loss
OAGB: one-anastomosis gastric bypass
SG: sleeve gastrectomy
AGB: adjustable gastric banding
JBI: Joanna Briggs Institute
